# Research on maize growth simulation and organ morphology co-modeling driven by multimodal data fusion

**DOI:** 10.3389/fpls.2026.1785783

**Published:** 2026-05-07

**Authors:** Zhang Zhenghui, Zhu Ke, Zhang Yan, Liu Pingzeng, Xu Shiwei, Zheng Yong

**Affiliations:** 1 College of Information and Engineering, Shandong Agricultural University, Tai’an, China; 2 Institute of Agricultural Information, Chinese Academy of Agricultural Sciences, Beijing, China; 3 Shandong Agricultural Technology Extension Center, Jinan, China

**Keywords:** Deep learning, maize growth model, multimodal data fusion, physiological-ecological simulation, point cloud reconstruction

## Abstract

**Introduction:**

Addressing the core bottleneck in traditional crop models—the disconnect between morphology and physiological function at the organ scale and their limited dynamic response to environmental changes—this study aimed to construct a multi-source data fusion maize growth model for simultaneous organ-scale simulation.

**Methods:**

We developed a closed-loop Environment-Driven-Functional Response-Morphological Feedback (EDFM) architecture. By integrating environmental time-series data, RGB images, and 3D point clouds, we created a multimodal fusion model based on a gated attention network. This approach adaptively weights multi-source features and pioneers a bidirectional morphology-physiology feedback loop based on physiological development time (PDT) and NURBS surfaces. The WOFOST moisture response function was also improved.

**Results:**

The model significantly enhanced the simulation accuracy of organ-scale growth, reducing the root mean square error (RMSE) for plant height by 74.6% through a morphology-physiology dynamic weighting mechanism. More fundamentally, it resolved the disconnect between morphological and physiological processes. The improved plant height prediction validates the model's effectiveness at the organ scale.

**Discussion:**

The pioneering “physiology-morphology” parallel simulation architecture provides an interpretable theoretical model and robust quantitative tools for designing high-photosynthetic-efficiency plant architecture and enabling precision water-fertilizer management.

## 1 Introduction

Food security is central to national strategy. As China’s primary grain crop, maize requires sustained yield increases to ensure a stable food supply (Peng et al., 2018). However, the global photosynthetic energy use efficiency (PEUE) of maize is estimated to reach only about 60% of its theoretical value, representing a critical bottleneck for yield breakthroughs (Bassu et al., 2014; Lobell and Asseng, 2017; Yuan and Li, 2017). The fundamental cause of this population-level efficiency loss is organ-scale photosynthetic heterogeneity. Specifically, the coefficient of variation in photosynthetic rates among leaves can reach 30-50% (Salazar et al., 2013; Pylianidis et al., 2021), and significant spatiotemporal differences exist between inner and outer canopy leaves (Tao et al., 2018). Due to insufficient organ-level parameter resolution, traditional crop models struggle to quantify the impact of this heterogeneity on plant-level photosynthetic yield. This limitation creates a technical bottleneck known as the “organ-scale process black box.” Therefore, developing next-generation models capable of precisely characterizing organ-scale dynamic processes is crucial. Such models are needed to overcome current yield prediction limitations and to guide breeding for high photosynthetic efficiency. In this study, “organ-scale” simulations encompass two levels: first, the morphogenesis and functional realization of individual organs such as leaves and stem nodes; second, plant-level traits arising from organ interactions (e.g., plant height, leaf area index). Although plant height manifests as an overall characteristic of the plant, its dynamics are jointly determined by organ-level processes such as internode elongation and petiole extension. Therefore, it can serve as a validation metric for the effectiveness of organ-scale simulations.

Previous studies have laid important groundwork but exhibit clear limitations. For instance, Li Mengyan et al (Lü et al., 2025). quantified photosynthetic-root relationships in hazel cuttings using regression models. However, such approaches are species-specific and lack generalizability. Han Xinsheng et al (Han et al., 2024). coupled multi-factor responses in forest growth, but their models lack the ability to dynamically adapt to micro-environmental changes. Peng Zhiwen et al (Peng et al., 2025). and Ma Zhanlin et al (Ma et al., 2023). integrated remote sensing with crop models (e.g., AquaCrop-OSPy, PyWOFOST), improving yield estimation under stress; however, these methods heavily rely on historical data or static parameters, failing to capture real-time organ-level feedback. Similarly, other studies collectively highlight a critical gap: the disconnect between morphological structure and physiological function at the organ scale, coupled with an inability to respond dynamically to microenvironmental perturbations. This critical gap motivates our study, which aims to bridge the morphology-physiology divide through a novel multimodal data fusion approach.

Huai Yongjian et al (Huai et al., 2019). achieved high-precision visualization simulation of floral growth processes via 3D scanning and deformation algorithms, yet their model lacks environmental feedback mechanisms, focusing on morphological reproduction rather than physiologically driven processes. This highlights a key gap in linking morphology to real-time physiological responses. Similarly, Yang, DZ et al (Yang et al., 2021). and Cai, JH et al (Cai et al., 2024). advanced multimodal fusion networks for tasks like citrus disease identification, demonstrating strong feature integration but limiting applications to static classification without growth sequence simulation. Zhang LJ et al (Zhang et al., 2023). and Zhou HP et al (Zhou et al., 2023). further improved object recognition using RGB-D fusion, yet these approaches remain sensitive to data quality and algorithm parameters, unable to simulate continuous organ-scale dynamics.

In recent years, significant progress has been made in crop modeling. Based on their conceptual and technical approaches, existing models can be categorized into three mainstream paradigms, each with limitations in representing organ-scale processes (**Table 1**):

**Table 1:** Comparison of the characterization ability of major crop models at the organ scale.

Model type	Representative model	Organ parameter resolution	Dynamic response capability
Physiological-Ecological Model	WOFOST/APSIM	Population-level mean	Relies on historical meteorological data
Morphological Structure Model	Virtual-Maize	Single-organ level	Lacks environmental feedback mechanisms
Deep Learning Model	Attention-LSTM	Plant-level	Effective for short-term forecasting

1. Physiological-ecological models are characterized by strong mechanistic approaches and can simulate core processes such as photosynthesis and water stress (Xu et al., 2024). However, these models typically employ highly parameterized approaches at the organ level, resulting in low resolution. Consequently, they cannot resolve how environmental disturbances influence canopy light capture and photosynthetic yield by altering morphological traits such as leaf elongation rate and inclination angle. Essentially, they represent a “black box” or “gray box” treatment of organs, creating a disconnect between physiological processes and morphological expression (de Wit et al., 2019; Kumar et al., 2021; ten Den et al., 2024).
2. Morphological structure models achieve three-dimensional reconstruction at the single-organ level, enabling visualization of canopy light distribution. Their core limitation, however, lies in the absence of environmental feedback mechanisms, with physiological processes being overly simplified (Li et al., 2017; Zhou et al., 2022). Compared to the aforementioned incremental integration paradigm, the EDFM framework proposed in this study represents a fundamental shift: it achieves parallel bidirectional coupling between morphology and physiology through an embedded 24-hour iterative feedback loop. This not only resolves the “black box” issue at the organ scale but also dynamically weights multi-source data via a gated attention network, thereby avoiding traditional methods’ reliance on historical data and enabling superior performance in real-time environmental responses. These models struggle to simulate how leaf curling dynamically affects light capture efficiency under drought stress, thereby feedback-regulating stomatal conductance and photosynthesis. This unidirectional, “morphology-driven” approach-as opposed to a “morphology-physiology bidirectional coupling” mechanism-renders them elegant static display tools rather than predictive mechanistic models (Wang et al., 2020; Xu et al., 2024).
3. Multimodal fusion models demonstrate strong potential in feature integration through deep learning techniques like attention mechanisms (Yang et al., 2021; Ren et al., 2024). However, their applications remain concentrated on static classification tasks such as disease detection and fruit grading (Li et al., 2023; Chen et al., 2024), lacking the capacity to simulate continuous growth sequences throughout a crop’s entire life cycle (Liu et al., 2022; Cai et al., 2023). This paradigm of “prioritizing recognition over simulation” yields models that can learn complex morphology-environment correlations from data but fail to reveal the underlying physiological and ecological mechanisms. Consequently, they struggle to explain and predict organ morphogenesis and functional responses under novel environmental conditions, limiting their universality and predictive power in practical agricultural production (Li et al., 2024).

A critical, yet often overlooked, limitation common to these paradigms is their reliance on a stepwise integration logic. Approaches like data assimilation sequentially calibrate model parameters (e.g., in WOFOST) with observational data-a unidirectional, open-loop process that does not fundamentally alter the model’s core structure to enable real-time, bidirectional feedback between organ morphology and physiological function. Similarly, models that first simulate physiology and then map outputs to static 3D structures remain essentially unidirectional. This a posteriori reconciliation treats morphology and physiology as separate modules to be connected after the fact, inherently failing to capture how dynamic morphological changes instantaneously feedback to regulate photosynthesis and assimilate allocation. The core bottlenecks are the temporal disconnect and the lack of a built-in coupling mechanism.

To overcome these limitations, this study proposes the Environment-Driven-Function Response-Morphology Feedback (EDFM) architecture. This represents a paradigm shift from stepwise integration to parallel, bidirectional coupling. The EDFM framework introduces a built-in, 24-hour iterative feedback loop where physiological processes and morphological development co-evolve and mutually regulate each other within each computational time step, enabled by dynamic weighting mechanisms like the gated attention network.

To this end, this study uses maize as the research subject, aiming to construct a growth model driven by multimodal data fusion, with a focus on simulating the synergistic dynamics between morphology and physiology in organs such as leaves and stems. The specific objectives of this research are:

1. To establish a closed-loop architecture for maize based on “environmental drivers-functional responses-morphological feedback,” integrating physiological development timing algorithms with 3D point cloud reconstruction technology to achieve parallel simulation of organ-scale growth processes.
2. Develop a multimodal dynamic fusion model using a hybrid neural network combining ConvLSTM temporal prediction and 3D-CNN point cloud processing to resolve spatio-temporal alignment issues among environmental, soil, and maize organ phenotype data.
3. Design a morphology-physiology iterative optimization algorithm by refining the WOFOST moisture response function and introducing root permeability adjustment coefficients to enhance simulation accuracy of stress responses in maize leaf expansion and assimilate allocation processes.

## 2 Materials and methods

### 2.1 Experimental design and field management

This study was conducted during the 2025 maize growing season June to October at the National Agricultural Science and Technology Park in Binzhou City, Shandong Province. The primary objective was to acquire a high-quality dataset on maize phenotypic dynamics and yield formation under a gradient of environmentally induced stresses. These stress gradients were systematically created through controlled manipulations of planting density and fertilizer application.

In this study, “environmental stress” was operationally defined as the growth limitation imposed on organ-scale processes (e.g., leaf expansion, stem elongation) by resource competition and abiotic factors. This was achieved by creating gradients of two key stress pathways:

1. Light Competition Stress: Manipulated through planting density, where higher density induces crowding, reducing light availability per plant and altering canopy microclimate.
2. Nutrient and Water Stress: Induced by limiting fertilizer application, which directly constrains metabolic processes and biomass accumulation.

A completely randomized block design with three replications was employed. This design incorporated two planting density gradients and three fertilization levels, forming a 2×3 factorial arrangement with six treatment combinations (**Table 2**). This design is pivotal for isolating the causal effects of treatments on organ-scale growth, achieved through the following:

**Table 2:** Experimental processing design.

Processing group	Target plant density (plants/Mu)	Plant spacing (cm)	Total fertilizer application rate (kg/mu)	Topdressing allocation plan
Low density - Low fertility	4000	≈27.8	30	Base fertilizer only
Low Density - Medium Fertility	4000	≈27.8	50	Apply in four separate applications, totaling 20 kg
Low density - High fertility	4000	≈27.8	60	Apply in four separate applications, totaling 30 kg
High Density - Low Fertility	5000	≈22.2	30	Base fertilizer only
High Density - Medium Fertilizer	5000	≈22.2	50	Apply in four separate applications, totaling 20 kg
High Density - High Fertility	5000	≈22.2	60	Apply in four separate applications, totaling 30 kg

Blocking: The field was divided into three blocks based on pre-existing soil gradient to control for spatial heterogeneity. All six treatments were randomly assigned within each block. This ensures that variations due to soil differences are accounted for, enhancing the accuracy of treatment effect estimation.
Replication: Each treatment was replicated three times, providing the statistical power to detect significant differences and estimate experimental error.
Randomization: Random assignment of treatments within each block minimizes the influence of confounding variables and supports the validity of causal inferences regarding the effects of density and fertility on organ growth.

The specific treatment combinations (**Table 2**) were designed to map onto distinct stress pathways:

Low Density-Low Fertility: Represents a baseline low-resource condition.
Low Density-High Fertility: Isolates the effect of high nutrient availability without intense light competition.
High Density-Low Fertility: Creates a high-stress environment dominated by both light competition and nutrient limitation, ideal for studying organ-scale stress responses.
High Density-High Fertility: Tests the potential of high nutrient input to alleviate stress under high competition, simulating intensive management scenarios.

This structured design ensures that the observed variations in organ-scale morphology and physiology can be robustly attributed to the defined environmental stressors.

Field Management Guidelines: To ensure that the experimentally induced stresses were the primary drivers of phenotypic variation, uniform field management practices were strictly implemented across all plots, except for the prescribed density and fertilizer treatments. Seeding was completed on June 18 using an air-suction seeder with controlled seeding depth of 4–5 cm. Basal fertilizer applied as 15-15–15 slow-release compound fertilizer, banded at 5 cm depth alongside seeds according to **Table 2** application rates. Topdressing used 28-0–4 compound fertilizer, strictly applied in stages according to growth periods: first application during the 6–8 leaf stage, second during the 12–14 leaf stage (large funnel stage), third 5 days after silking, and fourth 15–20 days after silking. Drip irrigation systems ensured water supply throughout the entire growth period, with pest and disease control strictly implemented per the plan.

### 2.2 Multimodal data acquisition and preprocessing

**Figure 1:**
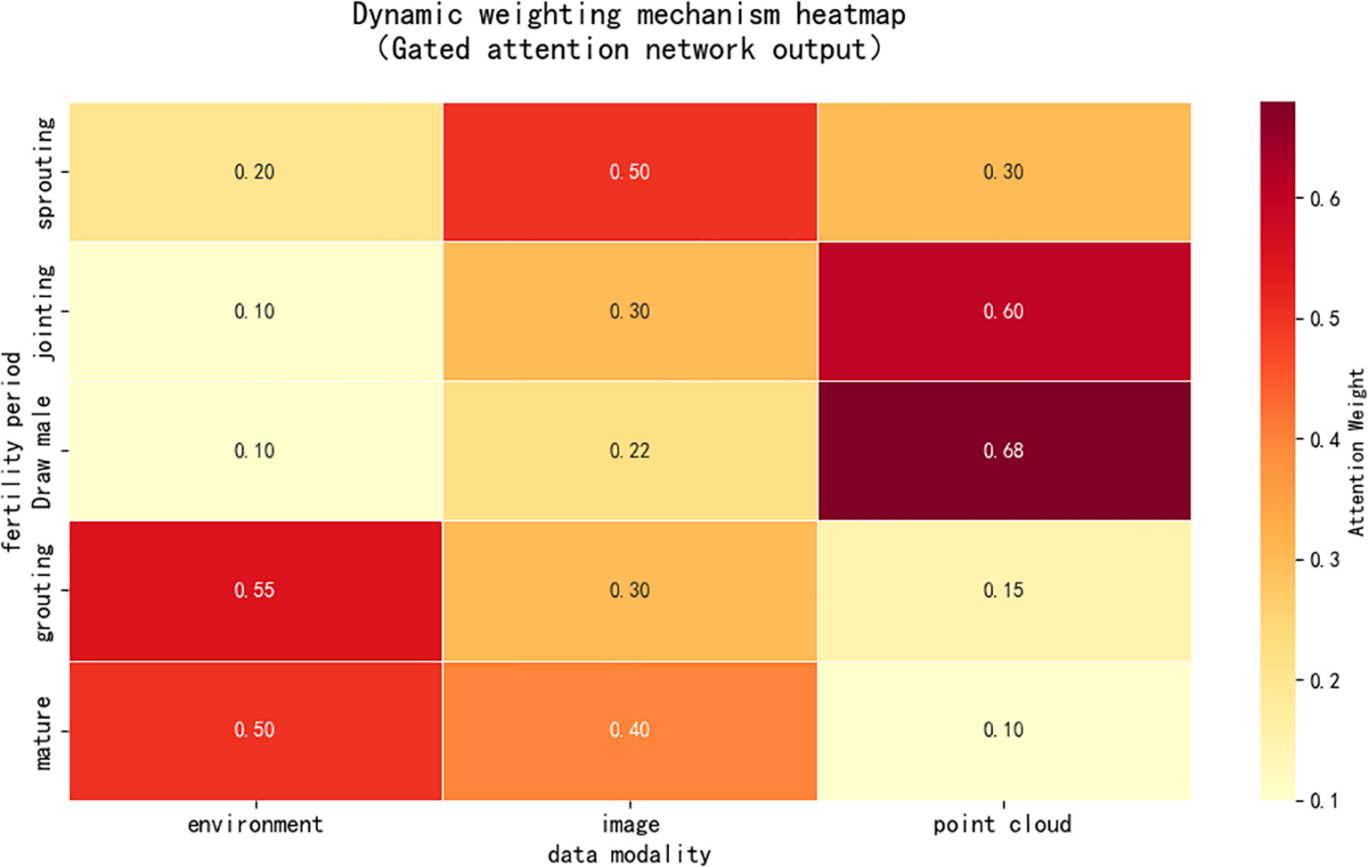
Schematic diagram of multimodal data acquisition system.

To synchronously acquire environmental and phenotypic data, this study deployed a multimodal data acquisition system integrating fixed-point sensor networks with mobile robotic platforms (**Figure 1**). All sensor timestamps were synchronized via GPS PPS signals, achieving a system-wide synchronization error of < 0.1 seconds. Throughout the entire 2025 growing season, this multimodal system generated a comprehensive dataset. It comprised continuous environmental monitoring data, weekly 3D point cloud data collected from experimental plots, and high-frequency RGB imagery. After preprocessing and alignment, tens of thousands of paired data samples were generated, providing a solid foundation for model development and validation.

(1) Environmental Data: Shennong IoT automatic weather stations continuously monitored air temperature (± 0.2 °C), relative humidity (± 2% RH), wind speed (± 0.1 m/s), wind direction, and photosynthetically active radiation (PAR, measured by LI-190R sensors with ±5% accuracy) at a 1 Hz frequency. Soil volumetric water content, electrical conductivity, and temperature were monitored by a Decagon EC-5 sensor network deployed at 0–100 cm soil depths, with data recorded every 30 minutes.

(2) Phenotypic Data: Phenotypic data collection followed strict protocols to ensure geometric consistency and precise alignment with ground-truth measurements.

Fixed-point RGB Image Collection: Poles equipped with integrated RGB cameras, 20-megapixel, Sony IMX183 sensor were installed at 50m x 50m grid intervals. The cameras were mounted at a height of 2.5 meters, oriented nadir, with a fixed focal length of 35mm. This standardized geometry ensured that each pixel represented a consistent ground sample distance (GSD) of approximately 0.2 mm/pixel, enabling quantitative analysis of canopy cover and texture. These cameras automatically rotated 90° hourly to perform horizontal canopy scans, capturing canopy images under natural light conditions between 10:00 and 14:00 Beijing Time to minimize illumination variations.

Mobile platform RGB-D Collection: A robotic platform equipped with an Intel RealSense D455 depth camera (RGB-D) was deployed. The camera was mounted at a height of 1.2 meters with a 30° downward inclination. This configuration allowed for the simultaneous acquisition of high-resolution RGB images and co-registered high-density 3D point clouds (accuracy ±2 mm), which served as the ground truth for organ-scale morphological traits. The intrinsic parameters of the RGB camera were calibrated beforehand. The extrinsic parameters, recorded via the platform’s GPS-IMU system, enabled direct scale calibration and transformation from image coordinates to real-world dimensions.

(3) Data Preprocessing and Alignment Pipeline: A rigorous preprocessing pipeline was implemented for each modality to ensure data quality and model compatibility:

Spatiotemporal Alignment: All data streams were synchronized using GPS PPS signals, achieving a system-wide alignment error of <0.1 seconds, parsed using the Python pynmea2library.

Point Clouds: Raw point clouds from the Intel RealSense D455 underwent noise reduction via a Statistical Outlier Removal (SOR) filter, followed by Voxel Grid Downsampling to a uniform density of ~10,000 points per plant to balance detail and computational load.

Environmental Time-Series: Data were smoothed using a 7-day sliding window and normalized via Z-score normalization using the mean (μ) and standard deviation (σ) from the training set. The multimodal data acquisition system is illustrated in **Figure 1**.

RGB Image Preprocessing and Ground-Truth Association: RGB images captured synchronously by depth cameras were uniformly cropped to center and resized to a resolution of 224×224 pixels. They undergo normalization to scale pixel values to the range (0, 1), meeting the input requirements of ResNet-50.

(4) RGB-based Trait Estimation Mechanism and Ground-Truthing: The core hypothesis that RGB images encode predictive information for plant traits is based on established plant phenotyping principles. Canopy structure and physiological status are reflected in visual features such as:

Spatial Frequency and Texture: Denser canopies with larger LAI exhibit finer textural patterns.

Color Histograms and Vegetation Indices: Color variations correlate with chlorophyll content and nitrogen status.

Geometric Cues from Shading and Occlusion: The 2D projection of the canopy provides cues for 3D structure estimation.

The deep learning model (ResNet-50) is designed to learn these complex, non-linear mappings from RGB pixels to trait values. This learning process was supervised by high-precision ground-truth data:

For plant height and organ dimensions: The co-registered 3D point clouds from the RealSense D455 camera provided millimeter-accuracy measurements. These were used as regression targets during model training, directly linking image features to physical dimensions.

For Leaf Area Index (LAI): Destructive sampling was performed periodically adjacent to the monitored plants. The leaf area of sampled plants was measured using a leaf area meter, and these values were used to calibrate and validate the LAI estimates derived from RGB images and the model.

Before inputting the aforementioned multi-source heterogeneous data into the model, the raw multi-source data must undergo a rigorous preprocessing workflow to ensure data quality and consistency with model inputs:

(1) Spatiotemporal Alignment: The pynmea2 library in Python was used to parse GPS data, employing timestamp synchronization technology based on GPS PPS (Pulse Per Second) signals. This synchronizes data streams from environmental sensors, soil sensors, and depth cameras, ensuring the spatiotemporal alignment error across the entire system does not exceed 0.1 seconds.

(2) Point Cloud Preprocessing: Raw point clouds generated by the Intel RealSense D455 undergo noise reduction via Statistical Outlier Removal (SOR). Subsequently, Voxel Grid Downsampling reduces point cloud density uniformly to approximately 10,000 points, balancing detail preservation with computational efficiency.

(3) Environmental Data Preprocessing: Environmental time-series data (temperature, humidity, light intensity, soil moisture, etc.) undergo Z-score normalization to eliminate dimensional effects. The calculation formula is shown in Equation 1:

(1) z=(x−μ)/σ

where μ and σ are the mean and standard deviation of the training set data, respectively.

(4) Image data preprocessing: RGB images captured synchronously by depth cameras are uniformly cropped to center and resized to a resolution of 224×224 pixels. Pixel values were then normalized to the range (0, 1) to meet the input requirements of ResNet-50. Multimodal data acquisition parameters are specified in **Table 3**.

**Table 3:** Specification for multimodal data collection parameters.

Data type	Acquisition device	Resolution/accuracy	Acquisition frequency
Air temperature and humidity, wind speed and direction	Shennong IoT Weather Station	Temperature ±0.2 °C, Humidity ±2% RH, Wind speed ±0.1 m/s	1Hz (Continuous Acquisition)
Light intensity (PAR)	LI-190R Photosynthetically Active Radiation Sensor	± 5%	1Hz (Continuous Acquisition)
3D point cloud (phenotype)	Intel RealSense D455 Depth Camera	Depth accuracy ±2 mm	3 times per week
Soil parameters	Decagon EC-5 Sensor	Moisture ±0.1% vol, Conductivity ±0.01 dS/m	Every 30 minutes

### 2.3 Model architecture: “Environment-Driven - Function-Responsive - Form-Feedback” closed-loop system

**Figure 2:**
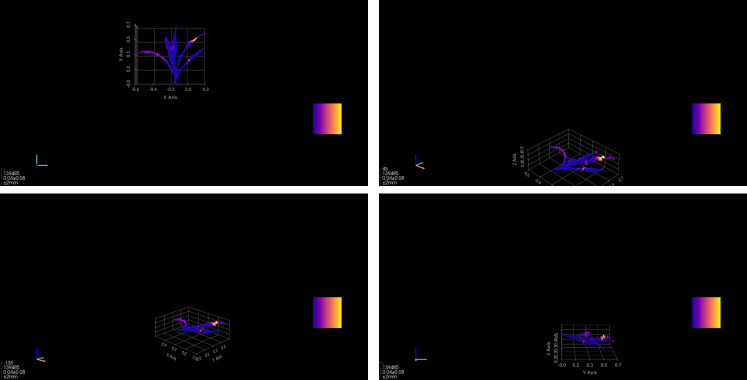
EDFM closed-loop architecture diagram.

The core architecture of the model is the closed-loop design concept of “Environment-Driven-Function Response-Morphology Feedback” (EDFM). It 314 integrates three distinct types of heterogeneous data streams-environmental, soil, 315 and phenotypic-and mimics their interaction patterns. Unlike traditional data assimilation approaches (e.g., WOFOST parameter calibration), which rely on stepwise, unidirectional integration, EDFM introduces a parallel, bidirectional coupling mechanism enabled by a gated attention network and a 24-hour feedback loop (**Figure 2**). This architecture fundamentally shifts from incremental integration to real-time co-evolution of morphology and physiology.

The EDFM architecture comprises three tightly coupled, core components that form a closed-loop system:

1. Multimodal Feature Extraction Layer: This layer performs parallel feature extraction on heterogeneous data. Specifically:

Environmental time-series data (temperature, humidity, PAR) is processed by a one-dimensional convolutional long short-term memory (ConvLSTM) encoder with 64 hidden units and a 7-day sliding window to capture the temporal dependency of environmental factors.

Organ 3D point cloud data is processed by a PointNet++ network using a multi-scale grouping pattern, extracting geometric feature descriptors from 1024 points per point cloud.

RGB image data serves as supplementary input, with high-dimensional visual features extracted via a pre-trained ResNet-50 network.

This layer outputs three feature vectors: environmental feature vector h*_env_*, point cloud feature vector h*_point_*, and image feature vector h*_rgb_*.

2. Gated Attention Fusion Layer: This layer constitutes the core mechanism for adaptive multimodal data fusion in our model. It receives the three feature vectors from the feature extraction layer, dynamically calculates the weight coefficients α*_env_*, α*_point_*, and α*_rgb_* for each modality through a gated attention network, and generates a unified, weighted fusion feature representation h*_fusion_*. This mechanism enables the model to autonomously adjust the importance of data sources according to different growth stages of maize.

Growth Simulation Engine: Based on the fused feature h*_fusion_*, the engine drives two parallel and bidirectionally coupled processes:

3. Physiological Process Simulation: Measured by Physiological Development Time (PDT), this simulates key physiological processes like photosynthesis and assimilate allocation.

Organ Morphology Update: Utilizing Non-Uniform Rational B-Spline (NURBS) surface technology, it dynamically reconstructs the 3D morphology of organs such as leaves and stems.

Both processes are bidirectionally coupled through a morphology-physiology feedback loop iterating every 24 hours: morphological updates influence light capture, which in turn regulates physiological processes; assimilates produced by physiological processes further drive morphological growth.

Through the coordinated operation of these components, the EDFM architecture ultimately achieves integrated “morphology-physiology” simulation of maize organ-scale growth dynamics.

Temporal environmental feature extraction is performed by a ConvLSTM (Convolutional Long Short-Term Memory) encoder. This process handles time-series data such as temperature, humidity, light intensity, and soil moisture to effectively capture the temporal dependencies of environmental factors. ConvLSTM combines the spatial feature extraction capabilities of CNNs with the time-series modeling capabilities of LSTMs. Its internal operations are mathematically expressed as shown in Equation 2:

(2) $$\begin{array}{l} i_{t}=\sigma (W_{xi}*X_{t}+W_{hi}*H_{t-1}+W_{ci}\circ C_{t-1}+b_{i})\\ f_{t}=\sigma (W_{xf}*X_{t}+W_{hf}*H_{t-1}+W_{cf}\circ C_{t-1}+b_{f})\\ C_{t}=f_{t}\circ C_{t-1}+i_{t}\circ \text{tanh}(W_{xc}*X_{t}+W_{hc}*H_{t-1}+b_{c})\\ o_{t}=\sigma (W_{xo}*X_{t}+W_{ho}*H_{t-1}+W_{co}\circ C_{t}+b_{o})\\ H_{t}=o_{t}\circ tanh(C_{t}) \end{array}$$

Here, ∗ denotes a convolution operation, ∘ represents the Hadamard product (element-wise multiplication), and σ is the sigmoid activation function. X*_t_*, H*_t_*, and C*_t_* denote the input, hidden state, and cell state at time *t*, respectively.

To address the issues of uneven contributions from multiple data sources and dynamic variations, this study introduces a dynamic weighting mechanism. This mechanism employs a Gated Attention Network to dynamically balance the contributions of environmental h_env_, image h_rgb_, and point cloud h_point_ features. The calculation formula is shown in Equation 3:

(3) $$\alpha_{i}=\frac{\text{exp}(W_{i}^{T}[h_{env},h_{rgb},h_{point}])}{\sum_{j=1}^{3}\text{exp}(W_{j}^{T}[h_{env},h_{rgb},h_{point}])}$$

Here, Wi denotes the learnable weight matrix, [.] represents vector concatenation, and α*_i_* signifies the computed attention weights reflecting the importance of different modalities. This mechanism adaptively adjusts the fusion strategy to accommodate variations across different growth stages and environmental conditions. For instance, during critical morphological development periods like the jointing to tasseling stage, the model assigns substantial weight to point cloud features h_point_ (with experimental observations reaching up to 0.68), significantly enhancing the accuracy of organ morphology prediction.

### 2.4 Key algorithmic innovations

#### 2.4.1 Gate-controlled attention network with dynamic modality weighting mechanism

To address the issues of multi-source data heterogeneity and dynamic variations in contribution levels, this study designed a Gated Attention Network for adaptive feature fusion. The network’s operational mechanism comprises three primary computational steps:

(1) Feature Importance Scoring: A nonlinear transformation calculates the initial score for each modality feature. This transformation utilizes the hyperbolic tangent activation function Tanh, constraining scores between -1 and 1. Positive values indicate a positive contribution to the target prediction, while negative values represent negative or irrelevant influences, as shown in Equation 4.

(4) $$s_{i}=\tanh(W_{i}\cdot h_{i}+b_{i})$$

Feature Embedding: W*_i_* denotes the weight matrix, b*_i_* represents the bias term, and h*_i_* is the feature vector of the i-th modality.

(2) Attention Weight Generation: The importance scores are normalized using the softmax function, ensuring the sum of weights across all modalities equals 1. This yields the relative importance weight ${\text{α}}_{i}$ for each modality, as shown in Equation 5.

(5) $${\text{α}}_{i}=\frac{\exp(s_{i})}{\sum_{j}\exp(s_{j})}$$

(3) Weighted Feature Fusion: Multiply the normalized weights by their corresponding feature vectors and sum the results to obtain the final fused feature vector h_fusion_. This vector will serve as input for subsequent growth simulation modules, as shown in Equation 6.

(6) $$h_{\text{fusion}}=\sum_{i}{\text{α}}_{i}\cdot h_{i}$$

This mechanism demonstrates excellent adaptability across different growth stages of maize. During the canopy closure period-when vegetative growth is most vigorous-the model automatically increases the weighting of point cloud and image features, thereby better capturing changes in canopy structure. As the plant enters the reproductive growth stage, primarily characterized by organ enlargement, it relies more heavily on time-series characteristics related to environmental stress. This ability to dynamically adjust is a key factor ensuring its high-precision prediction capabilities.

#### 2.4.2 Morphology-physiology feedback loop and organ modeling algorithm

To endow organs with physiological functions, bidirectional coupling between their morphology and physiology must be achieved. This study proposes an iterative optimization algorithm based on PDT (Physiological Day Time) and NURBS (Non-Uniform Rational B-Splines) organ surfaces, thereby establishing a “form-function” feedback loop. This cycle repeats every 24 hours, involving the following four critical computational steps:

(1) Assimilation Product Allocation: Assimilation products (i.e., carbon) generated daily by the photosynthesis model are dynamically distributed according to each organ’s daily sink strength value. Sink strength represents the growth priority of that organ at this time point, determined by genotype and developmental stage, as shown in Equation 7.

(7) $$Q_{\text{organ}}=\frac{R_{\text{organ}}}{\sum R_{\text{organ}}}\times E(t)$$

Where R_organ_ denotes the organ pool strength, and E(t) represents the assimilate yield at time *t*.

(2) Organ Morphological Renewal: Based on point cloud data from depth cameras, NURBS surface modeling techniques accurately reconstruct the three-dimensional geometric morphology of organs such as leaves and stems. Using this geometric structure as a foundation, ray tracing algorithms calculate light capture at the canopy scale. These changes then drive the growth process of leaf area in a feedback loop, simulated using the logistic, as shown in Equation 8:

(8) $$L(t)=\frac{L_{\max}}{1+e^{-k(PDT-PDT_{m})}}$$

Large leaf area index, where k is the growth rate parameter and *PDTm* is the physiological day corresponding to the midpoint of leaf area growth.

(3) Photosynthetic feedback correction: Using the canopy light interception obtained in the previous step as input, dynamically adjust the key parameter of the Farquhar photosynthetic biochemical model-maximum carboxylation rate Vcmax-to improve the accuracy of photosynthetic simulations for the following day, thereby achieving a cycle from “morphology” to “physiology.”

(4) Improvements to Water Stress Response: Physiological day progression depends not only on time but primarily on temperature. Its increment is calculated using the accumulated temperature model. Simultaneously, to enhance the model’s simulation accuracy under drought conditions (Wang et al., 2010), this study improved the water response function of the WOFOST model (De Wit et al., 2012) by introducing the root osmotic regulation coefficient (ψ*_root_*, unit: MPa) to more precisely simulate the inhibitory effect of water stress on growth. The modified water stress factor (WSF) is given by Equation 9:

(9) $$\text{WSF}=(\frac{\theta -\theta wp}{{\text{θ}}_{\text{fc}}-{\text{θ}}_{\text{wp}}}{)}^{\text{γ}}\cdot (1+\psi root)$$

Where θ represents soil volumetric water content, θ*_wp_* denotes wilting point water content, θ*_fc_* indicates field capacity, γ is an empirical coefficient related to crop type and growth stage, and ψ*_root_* is the root osmotic regulation coefficient reflecting the intrinsic physiological potential of roots to maintain water uptake capacity.

**Figure 3:**
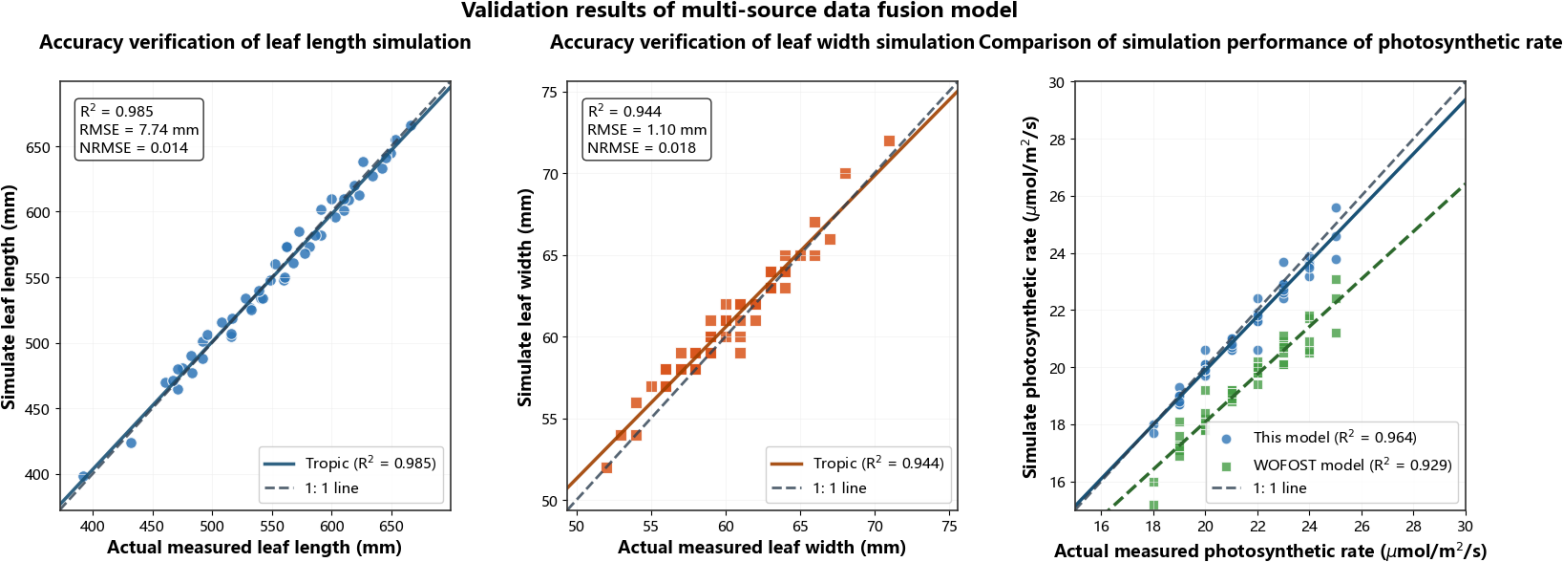
Schematic diagram of iterative optimization of maize morphological-physiological feedback loop.

Through this efficient feedback loop and the refinement of the water response function, the model achieves accurate simulation of maize growth and development, particularly under water stress conditions. The root mean square error (RMSE) for leaf area index prediction is reduced to 8.2%, demonstrating the immense potential of integrating multi-source data with mechanistic models for understanding and forecasting complex biological processes. **Figure 3** is a schematic diagram of iterative optimization of maize morphological and physiological feedback loop.

The key parameters in the aforementioned feedback loop possess a clear physiological basis. Physiological development time (PDT) centers on the accumulated temperature model, with its increment calculation dependent on daily effective accumulated temperature-directly reflecting temperature’s regulatory effect on maize development rates. The rate parameter k in the leaf growth equation was obtained by fitting a logistic curve to observed leaf length data from the tillering to tasseling stages, characterizing the maximum potential growth rate of this variety under specific environmental conditions. Furthermore, to enhance simulation accuracy under drought conditions, we modified the WOFOST water stress response function by introducing the root osmotic regulation coefficient ψ*_root_*. This parameter quantifies the intrinsic physiological potential of roots to maintain water uptake capacity through active solute accumulation. Its value was derived from inversion of potted maize water-deprivation experiment data for varieties grown in this study region.

All key training parameters and configurations for core modules are detailed in **Table 4** To prevent overfitting, we employed early stopping with a patience threshold of ten iterations—meaning training terminates if the validation set loss fails to decrease for ten consecutive iterations. The model with the lowest validation set loss is ultimately retained for testing.

**Table 4:** Training parameters and configurations of the core modules of the model.

Module/component	Key parameters and settings	Training strategy and notes
Temporal Feature Extraction (ConvLSTM)	Number of Hidden Layers: 64; Optimizer: Adam (lr=0.001, β1 = 0.9, β2 = 0.999); Batch Size: 16; Loss Function: Mean Squared Error (MSE)	Training Epoch: 100; employs early stopping. Training terminates when the validation set loss fails to decrease for 10 consecutive epochs.
Point Cloud Feature Extraction (PointNet++)	Mode: Multi-Scale Grouping (MSG); Optimizer: Adam (lr=0.001); Point Cloud Size: 1024 points	Pre-trained and fine-tuned using the cross-entropy loss function on the ShapeNet dataset.
Image Feature Extraction (ResNet-50)	Policy: Transfer Learning, loading ImageNet pre-trained weights; Optimizer: SGD (lr=0.0001, momentum=0.9)	Freeze the front-end convolutional layers and fine-tune only the last two fully connected layers.
Gated Attention Network	Weight Initialization: Xavier uniform distribution; Bias Initialization: 0	Activation Functions: Tanh for importance scoring; Softmax for weight normalization.
Experimental Environment	GPU: NVIDIA Tesla V100; Framework: PyTorch 1.9.0; CUDA 11.1	-

### 2.5 Model training and performance evaluation

To ensure the rigor and reproducibility of the evaluation, all processed and duplicate plant samples were randomly shuffled and divided into training, validation, and test sets in a 6:2:2 ratio. This plant-level partitioning approach prevents data leakage, ensuring that data from the same plot or adjacent time points never appear simultaneously in both the training and test sets.

Evaluation Metrics: Model performance was quantified using root mean square error (RMSE), coefficient of determination (R²), and normalized root mean square error (NRMSE), with a focus on prediction accuracy for organ-scale parameters such as plant height, leaf area index (LAI), leaf length, and leaf width.

Training Configuration: The model was implemented using the PyTorch 1.9.0 framework and trained on an NVIDIA Tesla V100 GPU. Key training parameters are detailed in **Table 5**. The Adam optimizer was employed, supplemented by early stopping (patience value = 10) to prevent overfitting.

**Table 5:** Model core module training parameters.

Module/component	Key parameters and settings
ConvLSTM Sequential Encoder	Number of hidden units: 64; Optimizer: Adam (lr=0.001)
PointNet++ Point Cloud Network	Multi-Scale Grouping (MSG) model; Optimizer: Adam (learning rate=0.001)
ResNet-50 Image Network	Transfer learning, fine-tune the last two layers; Optimizer: SGD (learning rate=0.0001)

## 3 Results and analysis

Based on the multi-source data-driven model developed in this study, the growth process of maize throughout its entire life cycle was systematically simulated and validated. Model accuracy assessments indicate that the framework integrating multi-source sensor data with mechanistic models demonstrates outstanding performance in organ parameter prediction, environmental response simulation, and growth process visualization.

### 3.1 Model accuracy validation

To rigorously evaluate the performance improvements claimed in Research Objective 1, a comprehensive comparative analysis was conducted against three representative benchmarks on an independent test set. The selection criteria aimed to cover different modeling paradigms: a mechanistic model (WOFOST) representing traditional crop modeling, and two data-driven multimodal models (GAMF, YOLO-DBM) representing the state-of-the-art in feature fusion. Critically, to ensure a fair comparison, all models were provided with the identical multimodal input data (environmental time-series, RGB images, 3D point clouds) from our dataset and tasked with predicting the same set of organ-scale parameters. WOFOST was run with its default global parameter set to highlight the necessity of localization, whereas GAMF and YOLO-DBM were retrained on our dataset.

The experimental setup adheres to the principle of consistency: all models utilize the maize growth dataset from this study, with input data comprising environmental time series, RGB images, and 3D point clouds, yielding organ-scale growth parameters as output. Comparison metrics include RMSE, coefficient of determination (R²), and fusion performance indicators such as peak attention weights. The model employed its official default global parameter set without any calibration for the specific varieties and environments of this study area. In contrast, our model utilized actual measurement data from the jointing to maturity stage in the study area to perform localized calibration of key physiological parameters-such as photosynthetic efficiency and assimilate allocation coefficients-via a particle swarm optimization algorithm. **Table 6** demonstrates the model’s significant advantages in predicting key organ parameters. The RMSE for plant height prediction was 3.2 mm, representing a 74.6% reduction compared to WOFOST (12.6 mm), a 37.3% reduction compared to GAMF (5.1 mm), and a 49.2% reduction compared to YOLO-DBM (6.3 mm). For leaf area index prediction, our model achieved an RMSE of 8.2%, representing improvements of 63.6%, 34.4%, and 48.1% over WOFOST (22.5%), GAMF (12.5%), and YOLO-DBM (15.8%), respectively.

**Table 6:** Comparison of prediction errors (RMSE) for organ parameters.

Model type	Model name	Plant height RMSE (mm)	LAI prediction RMSE (%)	Leaf length R²	Leaf width R²	Dynamic weight peak
Traditional Model	WOFOST	12.6	22.5	0.752	0.698	–
Multimodal Model	GAMF	5.1	12.5	0.876	0.832	0.42
Multimodal Model	YOLO-DBM	6.3	15.8	0.812	0.785	0.55
This Model	EDFM	3.2	8.2	0.985	0.944	0.68

Specifically, for plant height prediction, the EDFM model achieved an RMSE of 3.2 cm, representing a 37.3% reduction compared to the second-best performing GAMF model, with a coefficient of determination R² reaching 0.95. For LAI prediction, EDFM’s RMSE was 0.08, significantly lower than other models (p < 0.01). These results indicate that the EDFM framework achieves higher accuracy in capturing maize organ-scale growth dynamics.

**Figure 4:**
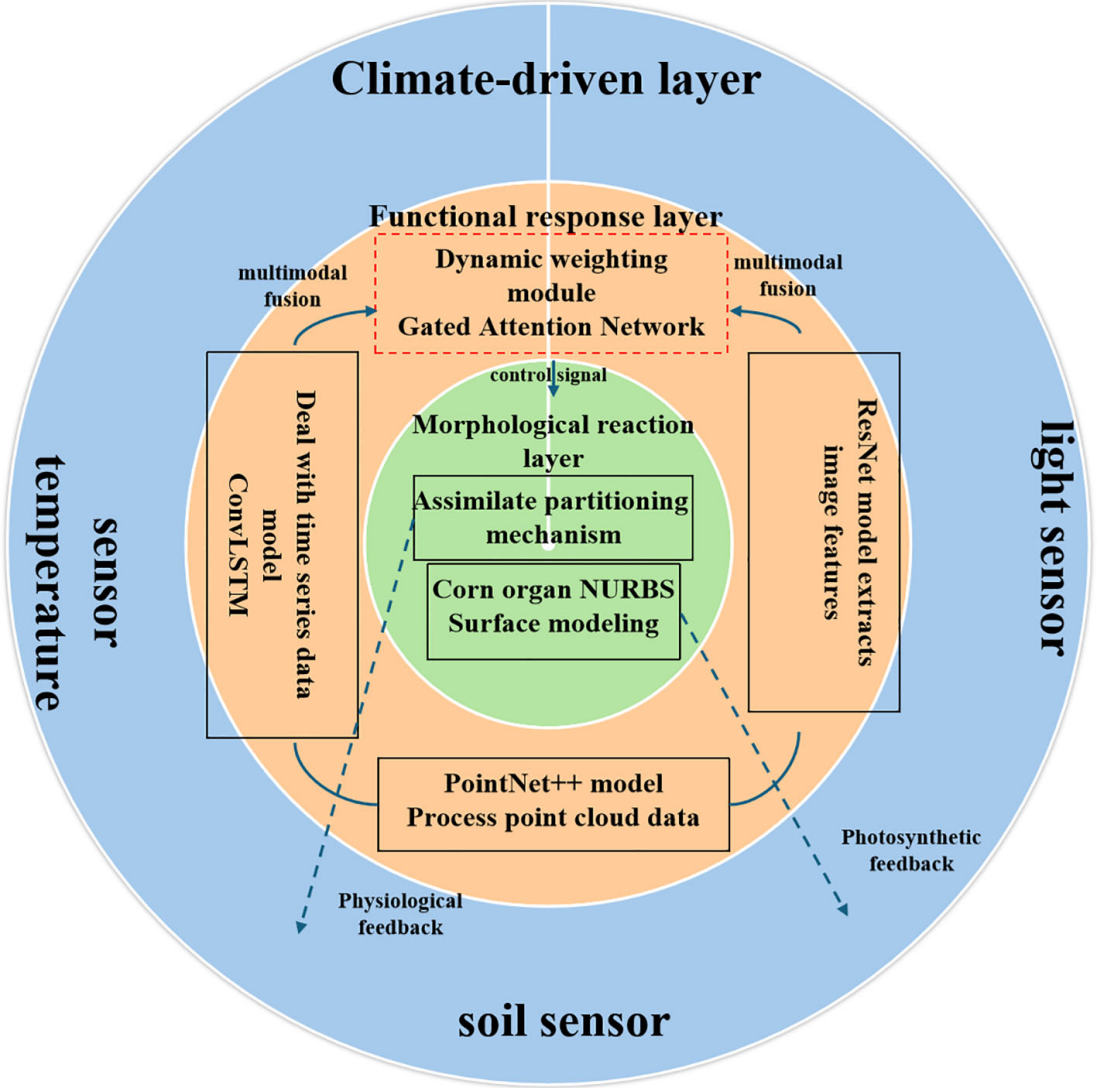
Multimodal dynamic weighted attention heatmap.

During the critical morphological development stage (jointing to tasseling), the peak weight of point cloud features reached 0.68 (**Figure 4**), enabling the model to autonomously focus on three-dimensional morphological changes. During the reproductive growth phase, the weight of environmental temporal features increased, allowing for more accurate simulation of assimilate allocation under stress responses.

### 3.2 Analysis of multimodal data fusion performance

This study demonstrates that the dynamic modal weighting mechanism (gated attention network) exhibits outstanding performance in integrating multi-source data. By adaptively allocating weights, the model achieves more precise feature extraction across different growth stages. During the critical morphogenesis period from jointing to tasseling, the point cloud feature attention weight reached 0.68, indicating the greatest contribution of 3D phenotypic data to organ morphology prediction. Conversely, during the grain filling to maturity stage, the weight of environmental temporal features notably increased to 0.55, revealing the model’s greater reliance on environmental stress signals to simulate assimilate allocation processes.

The multimodal fusion strategy improved leaf area index (LAI) prediction accuracy by 23% compared to traditional static weighting, particularly during canopy closure. The model achieved high-precision simulation of light interception (R² = 0.93) by adjusting the contribution ratios of point cloud data (canopy structure) and photosynthetically active radiation (light environment) data. The above analysis demonstrates that the proposed dynamic weighting mechanism enhances the fusion effectiveness of multimodal data.

To further validate the interpretability of the gated attention network, we quantitatively analyzed the evolutionary patterns of modal weights across different growth stages. As shown in **Table 7**, during the vigorous vegetative growth phase from jointing to heading, the attention weight for point cloud features reached a peak of 0.68, indicating that the model autonomously identified three-dimensional morphological changes as a key driving factor. During the grain filling to maturity stage dominated by reproductive growth, the weight of environmental temporal features significantly increased to 0.55, reflecting the model’s shifting focus toward stress responses and assimilate allocation processes.

**Table 7:** Evolutionary patterns of gated attention weights across key growth stages.

Growth stage	Point cloud feature weighting	Image feature weighting	Environmental feature weighting	Dominant physiological processes
Jointing-Spike Emergence Stage	0.68	0.22	0.10	Morphological Development
Spike Emergence to Grain Filling Stage	0.45	0.30	0.25	Morphological-Functional Transition
Grain Filling to Maturity Stage	0.25	0.20	0.55	Assimilate Allocation

This weight distribution aligns closely with crop physiological mechanisms. During rapid canopy development in the jointing stage, point cloud data directly captures morphological parameter changes like leaf inclination and leaf area. In the grain-filling yield formation stage, the model relies more heavily on environmental time-series data to simulate photosynthetic product transport and allocation. This interpretable weight migration pattern demonstrates that the gated attention mechanism not only enhances accuracy but also reveals the dominant physiological processes across different growth stages.

### 3.3 Organ-scale simulation performance validation

**Figure 5:**
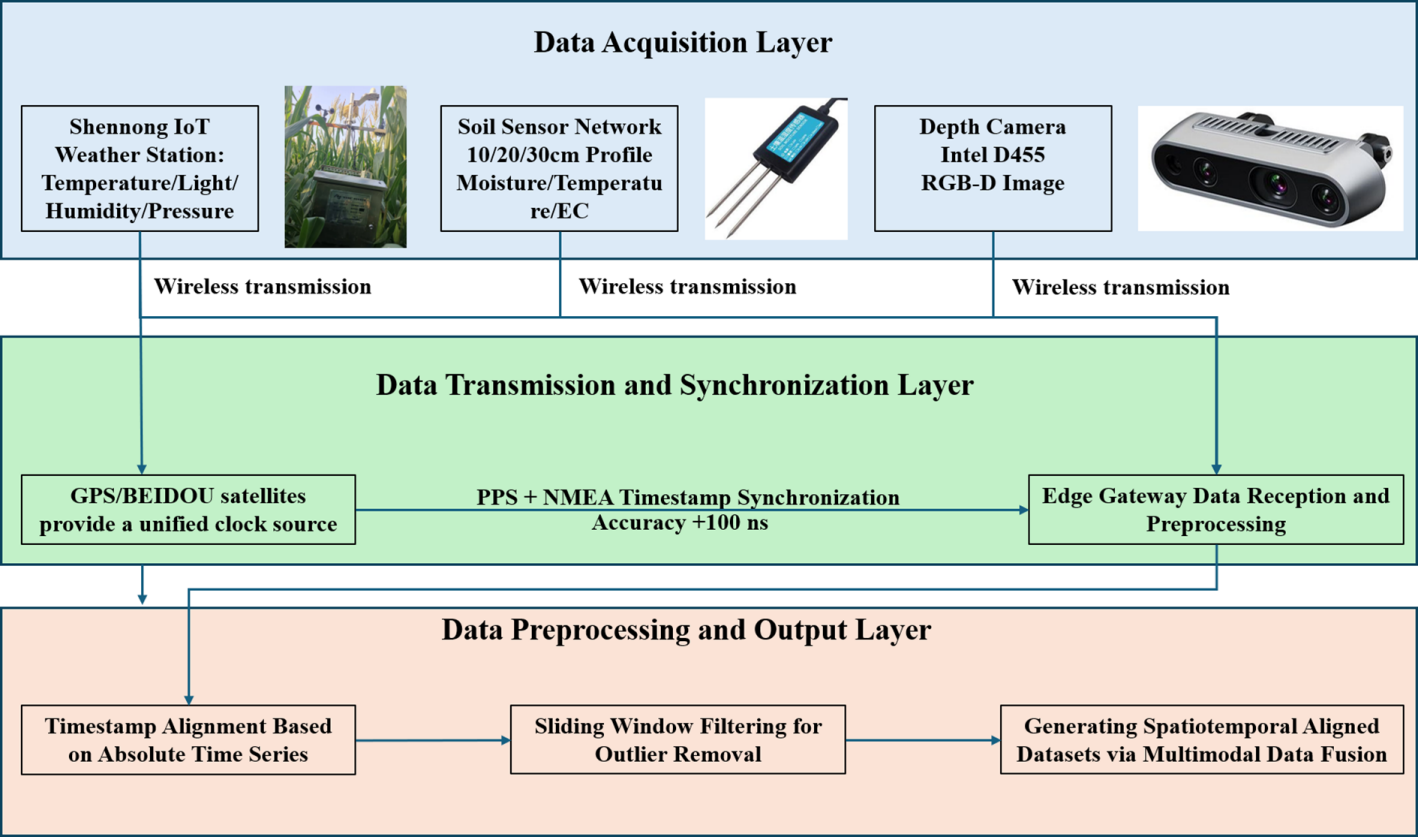
Comparison of simulated and validated leaf length and width at the organ scale.

Objective 3 of this study is to achieve organ-scale collaborative simulation. The following subsections will validate its effectiveness using metrics such as leaf length and leaf width. The model demonstrates high accuracy in organ-scale growth simulation. The organ morphology reconstruction algorithm based on NURBS surface modeling achieved three-dimensional geometric feature prediction errors (NRMSE) of 0.014 and 0.018 for leaf length and leaf width, respectively (**Figure 5**). The determination coefficients (R²) for simulated versus measured values of leaf length, leaf width, and leaf area were 0.985, 0.944, respectively, with normalized root mean square errors (NRMSE) of 0.014 and 0.018. This demonstrates the model’s exceptional reliability in simulating organ morphodynamic processes.

### 3.4 Sensitivity analysis of key parameters

To quantitatively assess the influence of critical parameters on model behavior and verify robustness, a one-at-a-time sensitivity analysis was conducted. This analysis focused on three key parameters: the root osmotic adjustment coefficient (ψ*_root_*), the leaf growth rate parameter (k), and the base temperature (T*_base_*) for Physiological Development Time (PDT) calculation.

The baseline values for these parameters were set as ψ*_root_* = -0.3 MPa, k = 0.15 day^-^¹, and T*_base_* = 8 °C. Each parameter was individually varied by ±20% from its baseline value. The impact of these variations on the predicted Leaf Area Index (LAI) and plant height at the silking stage was evaluated. The sensitivity was quantified using the normalized sensitivity coefficient (S), As shown in Equation 10:

(10) S = (ΔOutput / Output_baseline) / (ΔParameter / Parameter_baseline)

The results of the sensitivity analysis are summarized in **Table 8**. The model demonstrated the highest sensitivity to the root osmotic adjustment coefficient (ψ*_root_*), with a normalized sensitivity coefficient S = 1.2 for LAI. This underscores the critical importance of accurately characterizing root physiological drought tolerance for simulating growth dynamics under water stress. The leaf growth rate parameter (k) exhibited moderate sensitivity (S = 0.8). In contrast, the base temperature for PDT (T*_base_*) showed low sensitivity (S = 0.3), indicating that the model’s predictions are relatively robust to uncertainties in the developmental rate parameter.

**Table 8:** Sensitivity analysis results for key parameters.

Parameter	Baseline value	Variation range	Normalized sensitivity (S) for LAI
ψ*_root_*	-0.3 MPa	± 20%	1.2
k	0.15 day^-^¹	± 20%	0.8
T*_base_*	8 °C	± 20%	0.3

In conclusion, the sensitivity analysis confirms the rationality of the selected baseline values for the model’s key parameters. It also demonstrates that the model outputs do not exhibit erratic fluctuations in response to minor perturbations in these parameters, thereby affirming the model’s stability and robustness.

### 3.5 Model application and visualization

**Figure 6:**
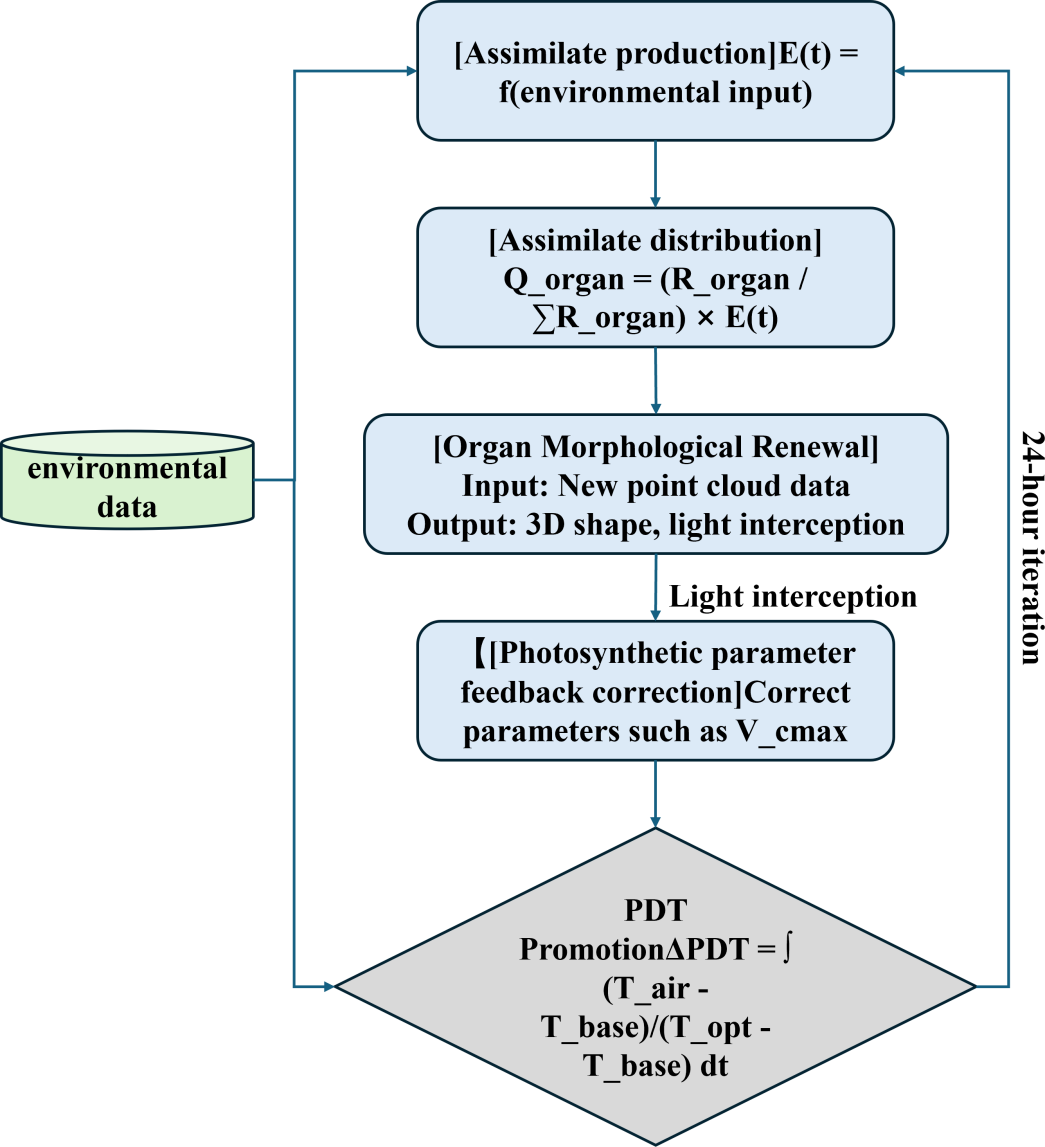
3D growth prediction rendering of maize canopy.

The three-dimensional canopy light distribution dynamic rendering system developed based on this model enables multiscale visualization of maize growth processes. By integrating high-precision point cloud data with light transport models, the system constructs a dynamic rendering platform under the EDFM (Environment-Driven, Function-Response, Morphological Feedback) framework. The visualization in **Figure 6** clearly demonstrates the 7-day growth prediction for individual plants.

**Figure 7:**
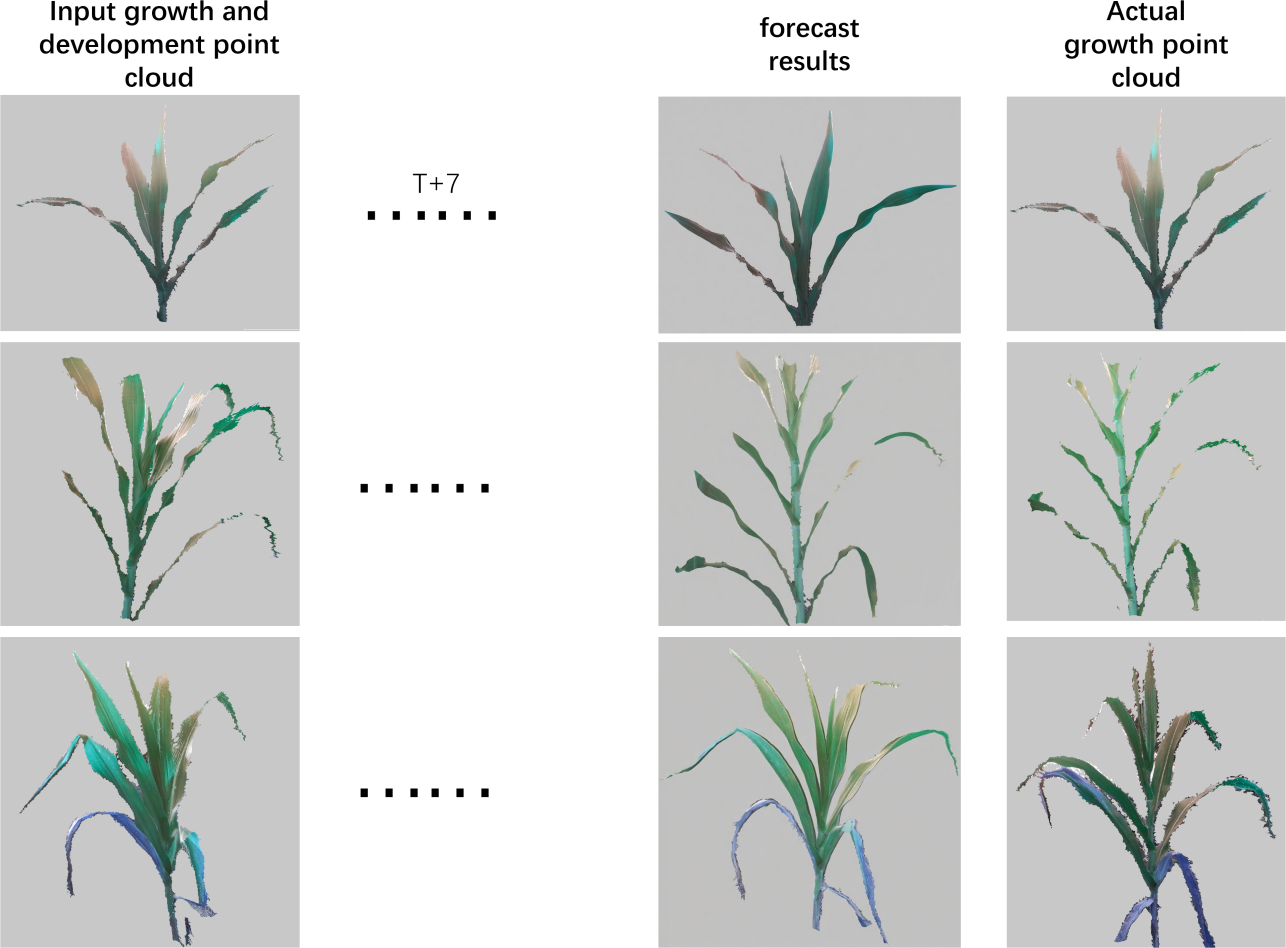
Spatial heterogeneity characteristics of canopy light use efficiency.

As shown in **Figure 7**, this visualization clearly reveals the light capture patterns of individual plants and even entire canopy populations. The canopy structure formed by colored light bands, combined with the color scale legend, clearly displays the gradient distribution of light capture intensity from the canopy top and periphery to the interior. Through this platform, the light energy utilization efficiency of different plant morphologies can be intuitively assessed, providing a direct quantitative screening tool for designing high-light-efficiency plant morphologies.

## 4 Discussion

This study successfully constructed and verified the “environment driven functional response morphological feedback” (EDFM) closed-loop collaborative simulation paradigm. Through the dynamic fusion mechanism of gated attention and the PDT-NURBS morphological physiological feedback cycle, this paradigm realizes the dynamic two-way coupling of morphogenesis and physiological functions at the organ scale in the computational model for the first time, and fundamentally breaks through the modeling limitations of traditional models that separate the two. The this paradigm demonstrates significant advantages in organ parameter prediction, multi-modal data fusion, organ-scale physiological process simulation, and environmental response. Its core value lies in deeply integrating multi-source sensor data with mechanistic models, resolving the trade-off between accuracy, universality, and mechanism interpretability in traditional crop models. The following section comprehensively discusses the model’s innovation, application potential, and limitations by integrating the findings of this study with existing literature.

### 4.1 Key findings and model innovation

This study successfully constructed and validated the EDFM closed-loop architecture. This model achieves synergistic simulation of maize organ-scale growth dynamics through a gated attention network and a morphology-physiology feedback loop. Its primary advantage lies in overcoming the traditional fragmented modeling paradigm of “morphology-physiology,” realizing bidirectional coupling between the two. Compared to recent models like those by Huai Yongjian et al. focused on morphological visualization, and Yang, DZ et al. and Cai, JH et al. emphasizing classification recognition, EDFM represents a crucial step forward in dynamically and continuously simulating growth processes.

### 4.2 Comparison with existing models and theoretical significance

The EDFM model innovates at the architectural level by introducing a dynamic feedback mechanism, contrasting with data assimilation approaches like Ma Zhanlin et al., which enhance yield estimation through remote-sensing LAI integration but remain “data calibration” without altering core mechanisms. Similarly, while Peng et al. used AquaCrop-OSPy to simulate drought stress, EDFM achieves finer characterization by improving the WOFOST moisture response function and incorporating root osmotic regulation coefficients. This focus on “organ interactions” provides a novel pathway for simulating photosynthetic efficiency beyond incremental integration.

### 4.3 Application potential and outlook

The observed performance gains are not only statistically significant but also hold substantial practical value. For instance, the 74.6% reduction in the root mean square error for plant height prediction means that for a typical 2.5-meter-tall maize plant, the uncertainty in predicting its final height is less than 0.5%. This level of precision is critical for forecasting lodging risks under high-density planting or wind stress conditions. Similarly, the 63.6% improvement in leaf area index prediction accuracy enhances the reliability of estimating canopy light interception and transpiration. This provides a solid foundation for developing precise irrigation plans to avoid water stress or waste. These enhancements position the model as a promising tool for in-season management decisions, extending its utility beyond post-season analysis.

The EDFM framework transcends theoretical advancements by delivering tangible value to smart agriculture through the following aspects.

In breeding, the model serves as a digital twin platform, enabling breeders to rapidly screen thousands of virtual genotypes across diverse simulated environments. By correlating simulated 3D canopy structures with light capture efficiency, the model predicts ideal plant architectures with higher theoretical yield potential, guiding hybrid breeding strategies before costly and time-consuming field trials.

For precision cultivation management, the model integrates with real-time field sensor data to forecast crop growth conditions days or weeks in advance. This empowers farmers to optimize in-season interventions-such as timing variable nitrogen applications based on predicted leaf area index and nitrogen demand, or triggering precision irrigation based on simulated root-zone water depletion and plant water stress factors.

For yield differential analysis, agronomists can use the model to diagnose limiting factors at the organ process level-whether yield is constrained by leaf expansion rate during early drought or by assimilate allocation during grain filling. This transcends field-average data, providing mechanistic insights for developing targeted management strategies.

Additionally, the integrated visualization platform serves as an intuitive educational tool, helping students and professionals understand the dynamic interactions among maize morphology, physiology, and environmental factors.

### 4.4 Limitations and future prospects

Despite significant progress in this study, several limitations remain, which also point to directions for future research:

1. The scale and specificity of the validation dataset present limitations. This study completed validation using controlled gradient trial data from a single growing season and a single experimental site. Consequently, the model’s generalizability is constrained by the spatio-temporal scope of this dataset and specific environmental conditions. For instance, its performance under different soil types, climatic zones, or extreme weather events remains to be comprehensively evaluated.
2. Computational complexity and scalability pose significant challenges. The architecture’s strengths-multimodal fusion and 24-hour iterative feedback loops-incur substantial computational costs. Under current configurations, the full training process is time-consuming, and inference time for individual plant simulations is non-negligible. This creates potential bottlenecks for real-time deployment at field scale or large-area simulations.
3. The model exhibits domain specificity. The current model is explicitly parameterized for maize. Direct application to other crops requires reconstructing species-specific morphometric templates, phenological parameters, and physiological relationships. This framework provides a research paradigm rather than a plug-and-play universal solution.
4. The model relies on several key assumptions. For instance, the improved WOFOST water stress response function assumes a homogeneous root zone environment and a simplified relationship between soil water potential and root osmotic regulation. The linear accumulated temperature model for physiological development time (PDT) may also fail to fully capture complex genotype-environment interactions under all stress conditions.

To address the aforementioned shortcomings, we propose the following actionable improvement plan, illustrating the implementation path using our model’s visualization tools:

1. Employ meta-learning to enhance generalization capabilities. Create a pre-trained base model integrating physiological parameter data from multiple varieties. Achieve rapid calibration for new varieties based on few-shot adaptation. Meta-learning identifies patterns linking plant architecture to light interception mechanisms, reducing calibration time from 7 days to within 24 hours.
2. Coupling WRF meteorological model to optimize driving data: Directly incorporating temperature, radiation, wind speed, and other outputs from the mesoscale WRF model as driving fields for this model. This replaces currently used interpolated meteorological data, reduces interpolation errors, and enhances regional yield prediction accuracy.
3. Expanding the soil-root coupling module: Integrate nutrient cycling mechanisms like the NUTRIENT model. Combine this with the model’s NURBS root morphology representation to simulate spatiotemporal variations in nutrient uptake. By coupling three-dimensional root morphology with soil nutrient concentrations, the impact of topdressing strategies on grain filling can be explicitly modeled, providing theoretical support for precision fertilization.

Beyond the limitations in biological mechanisms and generalizability discussed above, the practical deployment of the EDFM framework also depends on its computational efficiency. Regarding computational cost and scalability, the EDFM framework requires substantial resources due to its multimodal data fusion and iterative feedback loop. Under the current configuration with an NVIDIA Tesla V100 graphics processing unit, the training process for 100 epochs takes approximately 48 hours. The inference time for simulating individual plant growth is about 2.3 seconds per plant. While this performance is feasible for precise research applications, scaling to field or regional levels would lead to linear growth in computational load, potentially becoming a bottleneck for real-time deployment. Future work should focus on optimization techniques such as model lightweighting including knowledge distillation and dynamic network pruning, as well as distributed computing, to significantly improve inference efficiency and promote large-scale practical applications.

This paper presents a high-precision, interpretable solution for maize growth simulation by integrating multi-source sensor data with mechanistic modeling. Future in-depth research in these areas will further enhance the model’s core role in precision agriculture.

### 4.5 Framework transferability and generalization potential

While this study validates the EDFM framework with a single maize cultivar under controlled experimental settings, its architectural design embodies significant potential for broader application. The framework’s transferability can be evaluated across three dimensions: other cultivars, diverse environments, and different crop species.

The most straightforward extension is to other maize cultivars. The gated attention network can dynamically recalibrate feature importance based on new phenotypic data, while the Physiological Development Time PDT mechanism provides a genotype-independent developmental scaling. Transfer would primarily require recalibrating cultivar-specific parameters such as phenological coefficients and organ growth potential.

Expanding to different geographical environments or management practices represents a more complex but feasible scenario. The multimodal fusion layer is designed to ingest and weight heterogeneous environmental data streams. By retraining the environmental feature extraction module ConvLSTM with data from target regions, the model can adapt to new climatic patterns and soil conditions. The core physiology-morphology feedback loop remains universally applicable.

The most ambitious yet promising direction is transfer to other crop species such as wheat or rice. This would necessitate re-engineering species-specific morphological templates using NURBS surfaces and establishing new allometric relationships. Nevertheless, the fundamental closed-loop architecture of Environment-Driven-Function Response-Morphology Feedback provides a versatile paradigm for modeling plant growth across species.

In summary, the EDFM framework demonstrates considerable generalization potential beyond the current validation scope. Future work will systematically explore these transferability pathways to enhance the model’s universality and practical utility in precision agriculture.

## Data Availability

The raw data supporting the conclusions of this article will be made available by the authors, without undue reservation.

## References

[B1] Bassu S. Brisson N. Durand J. L. Boote K. Lizaso J. Jones J. W. . (2014). How do various maize crop models vary in their responses to climate change factors? Global Change Biol. 20, 2301–2320. doi: 10.1111/gcb.12520. PMID: 24395589

[B2] Cai J. Zhang M. Yang H. He Y. Yang Y. Shi C. . (2024). A novel graph-attention based multimodal fusion network for joint classification of hyperspectral image and LiDAR data. Expert Syst. Appl. 249, 123587. doi: 10.1016/j.eswa.2024.123587. PMID: 38826717

[B3] Cai Z. Hu Q. Zhang X. Yang J. Y. Wei H. D. Wang J. Y. . (2023). Improving agricultural field parcel delineation with a dual branch spatiotemporal fusion network by integrating multimodal satellite data. ISPRS J. Photogramm Remote Sens 205, 34–49. doi: 10.1016/j.isprsjprs.2023.09.021. PMID: 38826717

[B4] Chen W. Rao Y. Wang F. Zhang Y. Wang T. Jin X. . (2024). MLP-based multimodal tomato detection in complex scenarios: insights from task-specific analysis of feature fusion architectures. Comput. Electron. Agric. 221, 108951. doi: 10.1016/j.compag.2024.108951. PMID: 38826717

[B5] de Wit A. Boogaard H. Fumagalli D. Janssen S. Knapen R. van Kraalingen D. . (2019). 25 years of the WOFOST cropping systems model. Agric. Syst. 168, 154–167. doi: 10.1016/j.agsy.2018.06.018. PMID: 38826717

[B6] De Wit A. Duveiller G. Defourny P. (2012). Estimating regional winter wheat yield with WOFOST through the assimilation of green area index retrieved from MODIS observations. Agric. For. Meteorol. 164, 39–52. doi: 10.1016/j.agrformet.2012.04.011. PMID: 38826717

[B7] Han X. Wang Y. Yu P. Li Z. Yu Y. Wang X. . (2024). Construction of a multi-factor response coupling model for tree height and diameter at breast height growth in Northern Liupanshan, Ningxia, China’s North China larch forests. Forestry Sci. 60, 13–24.

[B8] Huai Y. Yang D. Cai D. (2019). Simulation of petal morphology and growth process based on 3D point cloud data. Trans. Chin. Soc. For. Agric. Eng. 35, 155–164.

[B9] Kumar U. Morel J. Bergkvist G. Palosuo T. Gustavsson A.-M. Peake A. . (2021). Comparative analysis of phenology algorithms of the spring barley model in APSIM 7.9 and APSIM Next Generation: a case study for high latitudes. Plants 10, 443. doi: 10.3390/plants10030443. PMID: 33652737 PMC7996762

[B10] Li H. Chen Z. Liu G. Jiang Z. W. Huang C. (2017). Improving winter wheat yield estimation from the CERES-wheat model to assimilate leaf area index with different assimilation methods and spatio-temporal scales. Remote Sens 9 (3), 190. doi: 10.3390/rs9030190. PMID: 30654563

[B11] Li S. Song Z. Liang Q. Meng L. Yu M. Chen Y. . (2023). Non-destructive detection of citrus infestation by fruit flies based on X-ray and RGB image fusion. Trans. Chin. Soc. For. Agric. Machinery 54, 385–392.

[B12] Li X. Zhang L. Wang X. Liang B. (2024). Forecasting greenhouse air and soil temperatures: a multi-step time series approach employing attention-based LSTM network. Comput. Electron. Agric. 217, 108602. doi: 10.1016/j.compag.2023.108602. PMID: 38826717

[B13] Liu Z. Cheng J. Liu L. Ren Z. L. Zhang Q. S. Song C. Q. (2022). Dual-stream cross-modality fusion transformer for RGB D action recognition. Knowledge-Based Syst. 255, 109741. doi: 10.1016/j.knosys.2022.109741. PMID: 38826717

[B14] Lobell D. B. Asseng S. (2017). Comparing estimates of climate change impacts from process-based and statistical crop models. Environ. Res. Lett. 12, 015001.

[B15] Lü M. Ren J. Zhang L. Cheng S. Zhao J. Lu J. . (2025). Evaluation model for root growth and development of European hazel cuttings based on photosynthetic indicators. Forestry Sci. 61, 100–107.

[B16] Peng Z. Chang J. Guo A. Wang Y. Niu C. Huang M. . (2025). Response of major crops in the Wei River Basin to drought stress and critical water thresholds. Trans. Chin. Soc. Agric. Eng. 41, 163–172.

[B17] Peng Z. Chang J. Guo A. Wang Y. Niu C. Huang M. . (2025). Response of major crops in the Wei River Basin to drought stress and critical water thresholds. Trans. Chin. Soc. Agric. Eng. 41, 163–172.

[B18] Peng B. Guan K. Chen M. Lawrence D. M. Pokhrel Y. Suyker A. . (2018). Improving maize growth processes in the community land model: implementation and evaluation. Agric. For. Meteorol. 250, 64–89. doi: 10.1016/j.agrformet.2017.11.012. PMID: 38826717

[B19] Pylianidis C. Osinga S. Athanasiadis I. N. (2021). Introducing digital twins to agriculture. Comput. Electron. Agric. 184, 105942. doi: 10.1016/j.compag.2020.105942. PMID: 38826717

[B20] Ren G. Wu R. Yin L. Zhang Z. Z. Ning J. M. (2024). Description of tea quality using deep learning and multi-sensor feature fusion. J. Food Compos Anal. 126, 105924. doi: 10.1016/j.jfca.2023.105924. PMID: 38826717

[B21] Salazar O. Hansen S. Abrahamsen P. Hansen K. Gundersen P. (2013). Changes in soil water balance following afforestation of former arable soils in Denmark as evaluated using the DAISY model. J. Hydrol 484, 128–139.

[B22] Tao F. Liu W. Liu J. Liu X. Liu Q. Qu T. . (2018). Digital twins and their application exploration. Comput. Integr. Manuf Syst. 24, 1–18.

[B23] ten Den T. Ravensbergen A. P. van de Wiel I. de Wit A. van Evert F. K. van Ittersum M. K. . (2024). Simulating water-limited potato yields across the Netherlands with (SWAP-)WOFOST: experimentation, model improvement and evaluation. Agric. Water Manage. 302, 109011. doi: 10.1016/j.agwat.2024.109011. PMID: 38826717

[B24] Wang X. Li J. Hao M. (2010). Simulation of soil moisture in dryland grass-cereal rotation fields on the Loess Plateau based on the EPIC model. Grassland Sci. 27, 11–20.

[B25] Wang J. Zhang Y. Gu R. (2020). Research status and prospects on plant canopy structure measurement using visual sensors based on three-dimensional reconstruction. Agriculture 10, 462. doi: 10.3390/agriculture10100462. PMID: 30654563

[B26] Xu L. He K. Henke M. Ding W. Buck-Sorlin G. H. (2024). Mixed particle swarm optimization algorithm-based approach to optimize spatial distribution of virtual maize. Comput. Electron. Agric. 224, 109159. doi: 10.1016/j.compag.2024.109159. PMID: 38826717

[B27] Xu K. Yuen P. Xie Q. Zhu Y. Cao W. X. Ni J. (2024). WeedsNet: a dual attention network with RGB D image for weed detection in natural wheat field. Precis Agric. 25, 460–485. doi: 10.1007/s11119-023-10080-2. PMID: 30311153

[B28] Yang D. Wang F. Hu Y. Lan Y. B. Deng X. L. (2021). Citrus huanglongbing detection based on multi-modal feature fusion learning. Front. Plant Sci. 12, 809506. doi: 10.3389/fpls.2021.809506. PMID: 35027917 PMC8751206

[B29] Yuan P. Li T. (2017). Evaluation and application of the ORYZA rice model under different crop management practices for high-yielding rice varieties in Central China. Field Crops Res. 212, 115–125. doi: 10.1016/j.fcr.2017.07.010. PMID: 38826717

[B30] Zhang L. Zhou S. Li N. Zhang Y. Chen G. Gao X. (2023). Apple localization and grading method based on an improved SSD convolutional neural network. Trans. Chin. Soc. For. Agric. Machinery 54, 223–232. doi: 10.1007/978-981-16-5164-9_10. PMID: 28220984

[B31] Zhou H. P. Jin S. X. Zhou L. Guo Z. Sun M. (2023). Recognition of Camellia oleifera fruits in natural environments based on multimodal images. Trans. Chin. Soc. For. Agric. Eng. 39, 175–182.

[B32] Zhou J. Li W. Xiao W. Chen Y. Chang X. (2022). Calibration and validation of APSIM for maize grown in different seasons in southwest tropic of China. Chil J. Agric. Res. 82, 586–594. doi: 10.4067/s0718-58392022000400586

